# Clinical Features and Prognostic Risk Prediction of Non-Hodgkin Lymphoma-Associated Hemophagocytic Syndrome

**DOI:** 10.3389/fonc.2021.788056

**Published:** 2021-12-06

**Authors:** Shuyan Yao, Zhili Jin, Lingbo He, Ruoxi Zhang, Menghan Liu, Zhengjie Hua, Zhao Wang, Yini Wang

**Affiliations:** ^1^ Department of Hematology, Beijing Friendship Hospital, Capital Medical University, Beijing, China; ^2^ Department of General Medicine, Beijing Friendship Hospital, Capital Medical University, Beijing, China

**Keywords:** hemophagocytic syndrome, non-Hodgkin’s lymphoma, cytokines, prognostic risk prediction, Epstein–Barr virus

## Abstract

**Background:**

Malignancies, especially lymphoma, are a common cause of adult secondary HLH and an independent risk factor for the prognosis of HLH patients.

**Methods:**

Patients with lymphoma alone or concurrent lymphoma-associated phagocytic syndrome (LAHS) admitted to Beijing Friendship Hospital from January 2016 to December 2020 were enrolled in this study.

**Findings:**

There were 348 lymphoma patients, 104 concurrent with LAHS. The pathological type of lymphoma without LAHS was dominated by B-cell lymphoma, while those with LAHS were T/NK-cell lymphoma predominantly (*p* < 0.001). Superficial lymph node enlargement was more significant in patients with B-LAHS (*p* = 0.006), while patients with T/NK-LAHS had lower neutrophil counts (*p* = 0.005), lower fibrinogen levels (*p* < 0.001), higher transaminase levels, and more co-infection with EBV (*p* < 0.001). B-LAHS had significantly higher IL-10 levels than with T/NK-LAHS (*p* = 0.006), and NK/T-LAHS had significantly higher IP-10 levels than other T-LAHS (*p* = 0.008). Age, platelet count, IPI, history of NK/T lymphoma, and no remission of HLH were independent risk factors for prognosis in patients with non-Hodgkin lymphoma-associated phagocytic syndrome (NHL-LAHS), and a prognostic risk score model for NHL-LAHS was developed.

**Conclusion:**

LAHS is a life-threatening disease with a poor prognosis. The prognostic risk score model for NHL-LAHS with a good fit and validation for the test has value for clinical application.

## Introduction

Hemophagocytic syndrome (HPS), also known as hemophagocytic lymphohistiocytosis (HLH), is a clinical syndrome resulting from inherited or acquired abnormalities of immune function (1). The disease was initially observed and studied in children, and subsequently found to be not rare in adults (2). The multifactorial analysis identified malignancy as an independent risk factor for the prognosis of patients with HLH, and lymphoma was the most common cause of malignancy-associated HLH (3). Due to the heterogeneous nature of HLH, a review of previous studies of lymphoma-associated HLH showed that the number of cases was generally small. Studies tended to focus on a subgroup of lymphomas, mostly NK/T lymphomas, and rarely tested the levels of soluble interleukin 2 receptors (sCD25) or the function of natural killer (NK) cells in adults (4–7). Therefore, this study aimed at demonstrating the clinical features of lymphoma-associated phagocytic syndromes (LAHS), establishing prognostic stratification, gaining a more comprehensive understanding of LAHS, and improving the overall prognosis of LAHS.

## Methods

### Patients

Patients with lymphoma alone or concurrent LAHS admitted to Beijing Friendship Hospital from January 2016 to December 2020 were enrolled in this study. It was approved by the Ethics Committee at Beijing Friendship Hospital. Informed written consent was obtained according to the Helsinki Declaration.

Lymphoma diagnosis was based on lymph node biopsy pathology concerning the World Health Organization classification of lymphoid neoplasms in 2016 (8). The staging of lymphoma was assessed using computed tomography (CT) or positron emission tomography (PET/CT) according to the Ann Arbor system, and the prognosis was assessed by the International Prognostic Index (IPI) score. HLH diagnosis was based on HLH-2004 diagnostic criteria (1). All patients with confirmed LAHS were screened for cytotoxic functions, including NK cell activity and degranulation function assays, and expression of proteins corresponding to HLH-deficient genes such as perforin, granzyme B, SAP, and XIAP. Gene sequencing should be performed to exclude primary HLH in patients with significant abnormalities in these conditions as well as in patients with recurrent LAHS.

We identified EBV infection by biopsy tissue ICH staining for EBER and real-time PCR of the patient’s blood, with at least one positive.

In addition to the above indicators mentioned, this study also examined the degranulation function (CD107a) and the levels of important cytokines such as IFN-γ and IL-10 to show the immune status in HLH patients.

### Follow-Up

Telephone follow-ups were conducted based on hospitalization information. Starting point: The day the patient was admitted to the hospital; endpoint: June 30, 2021, or the date of death or loss of follow-up.

### Statistical Analysis

The IBM SPSS Statistics 26.0 software was used. The measurement data were expressed as $\bar{\text{x}}$ ± sd or median (range); independent samples *t*-test, the one-way ANOVA test, the Mann–Whitney *U* test, and the Kruskal–Wallis test were used for comparisons, respectively. Count data were expressed as [cases (%)], and the *χ* ^2^ test was used for comparison between groups. Overall survival time was determined by the Kaplan–Meier method and assessed by log-rank test. The area under the receiver operating characteristic (ROC) curve is used to select bounds for valuable indicators. The prognostic risk score model was established according to the corresponding score for each risk factor (the regression coefficient of each independent predictor in the multivariate Cox proportional risk model was divided by the least regression coefficient, and the resulting value was rounded to the nearest whole number to be the risk score value of that factor). Hosmer–Lemeshow tests were also performed with the validation group to assess the fit. *p* < 0.05 was considered a statistically significant difference.

## Results

### General Characteristics

In total, there were 348 lymphoma patients, 104 concurrent with LAHS. Sixty-two LAHS patients in this study underwent HLH-related genetic testing and the results were negative. The general characteristics are shown in **Table 1**. The median age was 58 years for patients with lymphoma alone and 48 years for LAHS patients (*p* < 0.001); the pathological type of lymphoma without LAHS was predominantly B-cell lymphoma (179 cases, 73.4%), especially diffuse large B-cell lymphoma (DLBCL) (89 cases, 36.5%), and others included mainly follicular lymphoma (FL) (29 cases, 11.9%) and marginal zone lymphoma (MZL) (25 cases, 10.2%); T-cell lymphoma (31 cases, 12.7%) was mainly composed of angioimmunoblastic T-cell lymphoma (AITL) (12 cases, 4.9%), peripheral T-cell lymphoma not otherwise specified (PTCL-NOS) (9 cases 3.7%), and anaplastic large-cell lymphoma (ALCL) (7 cases, 2.9%). LAHS was dominated by T/NK-cell lymphoma (54 cases, 51.9%), especially NK/T-cell lymphoma (26 cases, 25%).

**Table 1:** Clinical characteristics of patients with lymphoma and LAHS.

	Lymphoma (*n* = 244)	LAHS (*n* = 104)	*p*-value
Age (years)	58 (16–90)	48 (13–81)	**<0.001**
Sex (M/F)	125/119	65/39	0.053
Pathology			**<0.001**
B	179	42	**<0.001**
DLBCL	89	29	**0.024**
FL	29	1	**0.019**
MZL	25	1	**0.036**
MCL	6	0	0.598
SLL	8	0	0.163
BL	6	0	0.598
LPL	3	0	1.000
others	13	11	**<0.001**
T	40	54	**<0.001**
PTCL-NOS	9	11	0.899
ALCL	7	5	0.276
AITL	12	3	**0.002**
NK/T	9	26	**0.007**
others	3	9	0.162
HL	25	5	0.098
cHL	18	4	1.000
Ann Arbor			**<0.001**
I/II	74	4	
III/IV	170	100	
IPI			**<0.001**
0–1	80	4	**<0.001**
2–3	138	79	**<0.001**
4–5	26	21	0.017
EBV (Y/N)	22/71	57/46	**<0.001**
CNS involvement	5	17	**<0.001**
ALT > 40 U/L	22	54	**<0.001**
AST > 40 U/L	27	60	**<0.001**
TIBL > 25 µmol/L	9	47	**<0.001**
ALB < 30 g/L	30	61	**<0.001**
LDH (U/L)	216 (93–3,750)	509 (79–5,601)	**<0.001**

DLBCL, diffuse large B-cell lymphoma; FL, follicular lymphoma; MZL, marginal zone lymphoma; SLL, small lymphocytic lymphoma; MCL, mantle cell lymphoma; BL, Burkitt lymphoma; LPL, plasma cell lymphoma; PTCL-NOS, peripheral T-cell lymphoma, not otherwise specified; ALCL, anaplastic large-cell lymphoma; AITL, angioimmunoblastic T-cell lymphoma; cHL, classic Hodgkin lymphoma; IPI, international prognostic index; EBV, Epstein–Barr virus; CNS, central nervous system; ALT, alanine transaminase; AST, aspartate transaminase; TBIL, total bilirubin; ALB, albumin; LDH, lactate dehydrogenase.

The bold values mean that they were statistically significant.

HLH was combined in 42 (19.0%) of 221 B-cell lymphomas (*p* < 0.001), 54 (57.4%) of 94 T/NK-cell lymphomas (*p* < 0.001), and 5 (16.7%) of 30 HL (*p* = 0.098). The Ann Arbor stage was III/IV in 170 (69.7%) lymphoma patients without LAHS versus 100 (96%) LAHS patients (*p* < 0.001); 218 (89.3%) lymphoma alone versus 83 (79.8%) LAHS had IPI scores less than 4 (*p* < 0.001). Compared with lymphoma alone, LAHS patients had significantly more co-infection with EBV (57, 55.3% vs. 22, 23.7%) (*p* < 0.001), central nervous system (CNS) involvement (17, 16.3% vs. 5, 2.0%) (*p* < 0.001), abnormal liver function (89, 85.6% vs. 57, 23.4%) (*p* < 0.001), and higher lactate dehydrogenase (LDH) levels (median 509 U/L vs. median 216 U/L) (*p* < 0.001).

Of all LAHS patients, 66 (63.5%) had HLH as the first presentation and 38 (36.5%) had lymphoma as the first presentation. There were only five HLs out of 104 LAHS patients, which was not representative enough, so this study focused on analyzing the clinical characteristics of NHL-LAHS shown in **Table 2**. The mean age of patients with T/NK-LAHS and B-LAHS was 41 and 52 years, respectively (*p* = 0.001). The superficial lymph node enlargement (40 cases, 95.2%) of patients with B-LAHS was more significant (*p* = 0.006) compared with T/NK-LAHS (40 cases, 74.1%), while patients with T/NK-LAHS had lower neutrophil count (median 1.3×10^9^/L vs. 2.2×10^9^/L) (*p* = 0.005), lower fibrinogen level (median 1.9 g/L vs. 3.3 g/L) and higher transaminase levels (ALT 59.5 U/L vs. 22.0 U/L, AST 60.2 U/L vs. 32.2 U/L) (*p* < 0.001) and more likely to have co-infection with EBV (41 cases, 75.9% vs. 8 cases, 19.5%) (*p* < 0.001).

**Table 2:** Clinical characteristics of patients with B-LAHS and T/NK-LAHS.

	B-LAHS (*n* = 42)	T/NK-LAHS (*n* = 54)	T-LAHS (*n* = 28)	NK/T-LAHS (*n* = 26)	Overall *p*	B vs. T^c^	B vs. NK^c^	T vs. NK^c^
Sex (M/F)	28/14	31/23			0.355			
Age (years)	52.31 ± 15.760	40.81 ± 15.704	43.11 ± 18.361	38.35 ± 12.103	**0.002**	0.018	**0.001**	0.269
IPI					0.090			
0–1	1	3						
2–3	28	44						
4–5	13	7						
Hepatomegaly	7	10			0.814			
Splenomegaly	39	44			0.106			
History of lymphoma	14	19			0.850			
Superficial lymph node enlargement	40	40	22	18	**0.012**	0.052	**0.005**	0.434
CNS involvement	4	12			0.098			
Fever	41	52			1.000			
Cytopenia^a^	20	25			0.897			
ANC (×10^9^/L)	2.24 (0.01–16.95)	1.25 (0.00–16.30)	1.345 (0–16.30)	1.05 (0.07–8.23)	**0.012**	0.054	**0.006**	0.283
Hb (g/L)	92.98 ± 23.115	93.13 ± 24.354			0.975			
PLT (×10^9^/L)	66.5 (5–343)	59 (2–944)			0.790			
FIB (g/L)	3.315 (1.20–8.68)	1.855 (0.35–8.28)	2.45 (0.35–6.19)	1.565 (0.44–8.28)	**<0.001**	0.030	**<0.001**	0.066
ALT (U/L)	22 (4–204)	59.5 (6–480)	50.5 (6–277)	84 (15–480)	**<0.001**	**0.006**	**<0.001**	0.077
AST (U/L)	32.2 (1.49–272.7)	60.2 (6.9–977.2)	54.55 (6.9–342.0)	83.9 (15.3–977.2)	**<0.001**	0.049	**<0.001**	0.104
ALB (g/L)	28.069 ± 5.4416	29.813 ± 4.7191			0.096			
TIBL (μmol/L)	22.68 (7.03–452.22)	22.725 (5.66–348.1)			0.570			
DIBL (μmol/L)	7.52 (1.72–176.05)	6.46 (0.86–193.57)			0.428			
IIBL (μmol/L)	15.235 (6.04–149.77)	15.45 (4.80–154.58)			0.679			
LDH (U/L)	597.5 (79–5,601)	496.5 (112–4,851)			0.828			
TG (mmol/L)	2.155 (0.65–72.00)	2.10 (0.79–8.00)			0.352			
Ferritin (μg/L)	1,438 (357.6–32,920.0)	2,529.2 (32–63,610)			0.166			
EBV (Y/N)	8/33	41/13	16/12	25/1	**<0.001**	**0.001**	**<0.001**	**0.001**
Decreased CD107a (Y/N)	8/26	18/29			0.270			
BM hemophagocytosis	31	37			0.572			
sCD25 (pg/ml)	33,374.5 (2,139–44,000)	20,613 (343–85,460)			0.385			
NK cell activity (%)	15.135 (8.66–42.58)	15.170 (2.73–46.61)			0.855			
IP-10^b^ (pg/ml)	95.15 (5–9,644)	124.8 (8–9,763)	56.40 (8–9,763)	275 (13–2,694)	**0.020**	0.491	0.025	**0.008**
IL-10^b^(pg/ml)	55.35 (1–12,745)	10.40 (0–7,051)	6.80 (0–5,841)	21.70 (0–7,051)	**0.008**	**0.006**	0.104	0.306
IL-1 RA^b^ (pg/ml)	553.18 (0–39,547)	204.30 (0–230,284)			0.368			
IFN-γ^b^ (pg/ml)	137.75 (1–1,731)	185.5 (0–1,971)			0.751			
IL-18^b^ (pg/ml)	182.93 (5–2,329)	190.6 (6–2,400)			0.710			
SDF-1α^b^(pg/ml)	532.95 (51–42,498)	406.9 (22–24,045)			0.435			
IL-22^b^ (pg/ml)	11.72 (3–3,742)	9.9 (0–23,391)			0.210			

^a^Cytopenia for at least two cell lines with hemoglobin <90 g/L, neutrophils <1.0 × 10^9^/L, and platelet <100 × 10^9^/L.

^b^Cytokine levels were tested in most patients with B-LAHS (n = 36) and T-LAHS (n = 47).

^c^p = α/k (α = 0.05, k = 3) is statistically significant.

ANC, absolute neutrophil count; Hb, hemoglobin; PLT, platelets; FIB, fibrinogen; DBIL, direct bilirubin; IBIL, indirect bilirubin; TG, triglyceride; BM, bone marrow; NK, natural killer.

The bold values mean that they were statistically significant.

The inflammatory state of HLH is often characterized by hypercytokinemia. In this study, except for IL-10, which was significantly higher in patients with B-LAHS (median 55.4 pg/ml) than in patients with T/NK-LAHS (median 10.4 pg/ml) (*p* = 0.008), no significant differences were seen for other cytokines.

Since NK/T-NHL is an important subtype of T-cell NHL, T/NK cell lymphoma was further subclassified as shown in **Table 2**. The difference between B-LAHS and T/NK-LAHS is mainly from B-LAHS and NK/T-LAHS. Regarding co-infection with EBV, there was a significant difference among B, NK/T-LAHS, and other T-LAHS (*p* < 0.001), with B-LAHS less frequently co-infected with EBV (8 cases, 19.5%), T-LAHS second (16 cases, 57.1%), and NK/T-LAHS most frequently co-infected with EBV (25 cases, 96.2%). About cytokine levels, comparing NK/T-LAHS with other T-LAHS, IP-10 levels were significantly higher in the former (275 vs. 56.4 pg/ml) (*p* = 0.008). Comparing B-LAHS with T-LAHS other than NK/T, the former had significantly higher IL-10 levels than the latter (55.4 vs. 6.8 pg/ml) (*p* = 0.006).

Furthermore, EBER DNA copies in plasma and peripheral blood mononuclear cells (PBMCs) were higher in patients with NK/T-LAHS than in other T-LAHS, but none of them was statistically significant (*p* > 0.05). Twenty-three cases were positive for EBER in NK/T-LAHS, 14 in the bone marrow, and 4 in lymph nodes, while 10 cases were positive in other T-LAHS and 7 in lymph nodes, with significant differences (*p* = 0.001).

### Treatment and Survival

**Figure 1:**
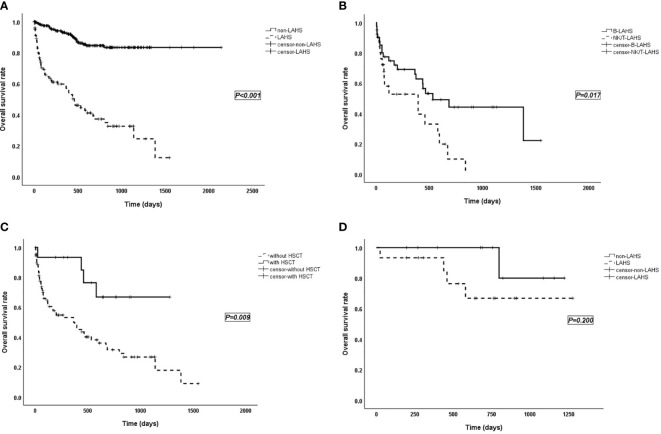
**(A)** Survival curves of patients with lymphoma without LAHS (*n* = 244) vs. with LAHS (*n* = 104). “Non-LAHS” means lymphoma without LAHS. **(B)** Survival curves of patients with B-LAHS (*n* = 42) vs. NK/T-LAHS (*n* = 26). **(C)** Survival curves of LAHS patients with HSCT (*n* = 15) vs. without HSCT (*n* = 89). **(D)** Survival curves of patients received HSCT with lymphoma alone (*n* = 11) vs. LAHS (*n* = 15). “Non-LAHS” means lymphoma alone.

Among the 348 lymphoma patients, the prognosis of patients with lymphoma alone was significantly better than that of LAHS patients (*p* < 0.001; **Figure 1A**).

The treatment regimen for HLH in this study was mostly based on DEP (doxorubicin-etoposide-methylprednisolone) (9) combined with chemotherapy. Patients were observed for treatment response, 2 in complete response (CR), 56 in partial response (PR), 30 in no response (NR), 3 relapsed, and 1 flared up. The median follow-up time for overall LAHS patients was 648 (range 4–1,548; 95% CI: 453–843) days, and median survival time was 459 (95% CI: 309–609) days. In different pathological types, the median survival time was 533 (range 5–1,548) days for B-LAHS patients, 468 (range 4–1,278) days for T-LAHS patients, and 395 (range 5–840) days for NK/T-LAHS patients. Overall survival (OS) rates at 0.5, 1, and 2 years were 72.0%, 66.0%, and 44.2% for B-LAHS patients and 52.8%, 52.8%, and 9.9% for NK/T-LAHS patients, respectively, with significant differences (*p* = 0.017; **Figure 1B**). OS rates for T-LAHS were 60.9%, 51.1%, and 46.0%, respectively, with no significant differences compared with the other two groups.

Of the 104 LAHS patients, 89 did not receive hematopoietic stem cell transplantation (HSCT) with a median survival time of 365 days, while 15 received HSCT (including 4 autologous and 11 allogeneic) with a mean survival time of 987 days (*p* = 0.009; **Figure 1C**). Eleven lymphoma patients without LAHS received HSCT, namely, nine autologous and two allogeneic (*p* = 0.200; **Figure 1D**).

### Prognostic Risk Score Modeling

There were 96 cases of NHL-LAHS from January 2016 to December 2020 in this study. We randomly took 16 cases from 2017 to 2019 to validate the risk model for the prognosis of patients with NHL-LAHS developed by the remaining (80 in total).

The results of the univariate analysis of patients with LAHS are presented in **Table 3**; NK/T pathology type, co-infection with EBV, and HLH in remission without HSCT were prognostic risk factors (*p* < 0.05). Age, IPI score, and platelet count were also prognostic risk factors by one-way ANOVA assay (*p* < 0.05).

**Table 3:** Cox regression analysis of prognostic risk factors.

Risk factor	Univariate model		Multivariate model		Prognostic model	
	HR (95% CI)	*p*	HR (95% CI)	*p*	*β*	Source
History of lymphoma (Y/N)	0.523 (0.256–1.069)	0.076				
PLT			0.989 (0.982–0.997)	**0.004**		
≤20 × 10^9^/L			3.009 (1.311–6.904)	**0.009**	1.102	2
20 < PLT < 60			2.240 (1.066–4.709)	**0.033**	0.807	1
≥60 × 10^9^/L						
Age			1.031 (1.002–1.062)	**0.039**		
>45 years			2.758 (1.338–5.685)	**0.006**	1.015	1
≤45 years						
IPI			1.520 (1.008–2.292)	**0.045**		
3–5			2.739 (1.251–5.994)	**0.012**	1.008	1
0–2						
Sex (M/F)	1.117 (0.821–1.519)	0.483				
NK/T (Y/N)						
Y	2.080 (1.104–3.921)	**0.023**	2.652 (1.258–5.592)	**0.010**	0.975	1
N						
EBV infection (Y/N)	2.005 (1.068–3.762)	**0.030**	0.644 (0.290–1.429)	0.279		
EBV DNA copies in plasma (>500/≤500)	1.664 (0.899–3.081)	0.105				
EBV DNA copies in PBMC (>500/≤500)	1.616 (0.878–2.976)	0.123				
Superficial lymph node enlargement (Y/N)	1.068 (0.508–2.243)	0.863				
CNS involvement (Y/N)	0.793 (0.351–1.790)	0.576				
Fever (Y/N)	2.214 (0.303–16.158)	0.433				
Splenomegaly (Y/N)	0.829 (0.349–1.971)	0.672				
HSCT (N/Y)	3.397 (1.046–11.024)	**0.042**	0.945 (0.246–3.631)	0.934		
Decreased CD107a (Y/N)	1.763 (0.967–3.212)	0.064				
HLH in remission^a^ (N/Y)						
N	2.579 (1.397–4.759)	**0.002**	2.003 (1.059–3.788)	**0.033**	0.695	1
Y						
BM hemophagocytosis (Y/N)	1.296 (0.670–2.509)	0.441				

^a^R and PR are considered as HLH remission; NR, relapses and exacerbations are not.

The bold values mean that they were statistically significant.

The seven risk factors obtained above were subjected to COX multifactorial analysis as shown in **Table 3**, and age, NK/T pathology type, PLT count, and IPI score with no remission were all independent risk factors for the prognosis of LAHS patients.

**Figure 2:**
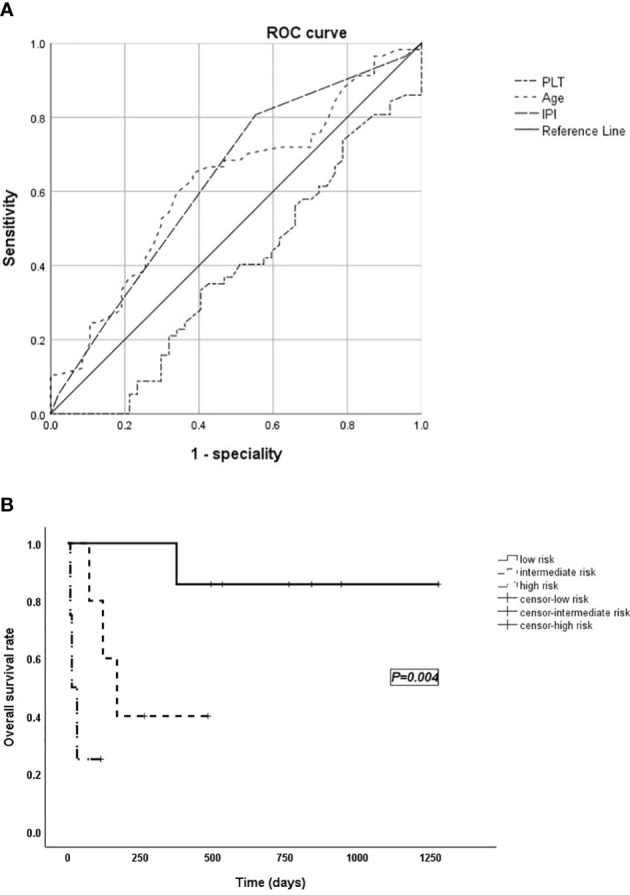
**(A)** The area under the receiver operating characteristic (ROC) curve used to select bounds for IPI, age, and PLT. **(B)** Survival curves of the validation group.

The area under the receiver operating characteristic (ROC) curve was used to select bounds for IPI, age, and PLT as shown in **Figure 2A** (area for PLT: 0.385, *p* = 0.044; area for age: 0.619, *p* = 0.037; area for IPI: 0.632, *p* = 0.021), and the NHL-LAHS prognostic risk score model is shown in **Table 3**.

The Hosmer–Lemeshow test (*p* = 1.000) indicated that the model fitted the data well. Based on the model, the validation group of cases was divided into groups: low risk (0–1 score), intermediate risk (2–3 score), and high risk (4–5 score) with a significant difference in survival time between the three groups (*p* = 0.004; **Figure 2B**).

## Discussion

Tumor-associated HLH is known to occur during the treatment of malignancy or to manifest when malignancy has not yet been diagnosed, and the pathogenesis is tumor stimulation, immune suppression by chemotherapy, genetic, and infection-triggered (9). In this study, 38 (36.5%) patients with HLH had a history of lymphoma, while 66 (63.5%) with HLH as their first presentation developed or discovered lymphoma. That is, HLH may mask the underlying lymphoma of patients, making the diagnosis of lymphoma difficult. In China, NK/T-cell lymphoma is the predominant subtype of LAHS (10), and in Japan, although DLBCL is the most common pathological type of LAHS, the incidence of concurrent LAHS is higher in patients with T/NK-cell lymphoma than in B-cell lymphoma (11). This study found similarly that T/NK-cell lymphoma, especially NK/T-cell lymphoma, was more likely to be combined with HLH compared with B-cell lymphoma (*p* < 0.001). Regarding infection, only 22 lymphoma patients tested positive for EBV (23.7%), while 57 (55.3%) LAHS patients were combined with EBV infection (*p* < 0.001). Among these, 8 (19.5%) had EBV infection in B-LAHS, 16 (57.1%) in T-LAHS, and 25 (96.2%) in NK/T-LAHS (*p* < 0.001). However, an in-depth analysis of patients with HL-LAHS was not performed because there were only five HLs in the overall LAHS. Classical Hodgkin lymphoma complicated by HLH is rare relative to other lymphomas, but Mánard et al. found a strong correlation with EBV in 34 patients with HL-LAHS, considering some link between it and X-linked lymphoproliferative disease (12). EBV has a pathogenic role in the development of extranodal NK/T-cell lymphoma nasal type (ENKTL-NT), and circulating EBV-DNA copies reflect tumor load and activity (13). In this study, the median EBV-DNA copies in plasma and PBMCs of patients with NK/T-LAHS were 170,000 and 11,000, respectively, higher than those in other T-LAHS (24,000 and 6,200), but the differences were not statistically significant (*p* = 0.531 and 0.804). Interestingly, the result of a study demonstrated that EBV-DNA titers detected in the plasma of patients with extranodal NK/T-cell lymphoma (ENKTL) at more than 4,450 copies/ml was a predictor for NK/T-LAHS (14). Remarkably, up to 10% of all DLBCLs, as well as all cases of endemic BL are now recognized as being EBV-positive, while only 10%–15% of sporadic BL are EBV-positive (15). However, Jin et al. identified the lineage of EBV-infected cells from EBV-HLH patients by immunohistochemistry double staining and found that most of them were CD31/EBER1, suggesting that EBV predominantly infects T cells in Chinese populations (16). It supports the idea that NHL-LAHS was more frequently associated with EBV infection than NHL and T/NK-LAHS than B-LAHS in this study.

The gold standard for detecting EBV remains *in situ* hybridization (ISH) of EBV-encoding RNA (EBER) in biopsied tissue (17). A study of 62 lymphomas found that 31.3% of PTCL-NOS and 100% of ENKTL-NT were positive for EBER in tumor cells (18). In this study, 23 (88.5%) NK/T-LAHS and 10 (35.7%) T-LAHS were EBER positive (*p* = 0.001), consistent with previous lymphoma studies. In addition, NK/T-cell lymphoma occurs mainly in extranodal organs, while primary nodal manifestations are very rare (19). In this study, there were 40 cases (95.2%) of superficial lymph node enlargement in patients with B-LAHS and only 18 cases (69.2%) in patients with NK/T-LAHS (*p* = 0.005).

The pathogenesis of primary HLH is known to be altered NK cell toxicity and reduced degranulation function due to genetic mutations. Corresponding functional screening tests can rapidly help in diagnosing and classifying patients with primary HLH (20). A multicenter study found that although NK cell activity and CD107a degranulation function assays are consistent, they are not interchangeable (21). A prospective multicenter study found that secondary HLH (sHLH) is not due to a primary cytotoxic defect but mimics other hyperinflammatory syndromes associated with cytokine storm (22). In this study, the median level of NK cell activity in patients with NHL-LAHS was similar to the lower limit of normal, and CD107a did not reduce in nearly 73% of patients, with no significant differences between B-LAHS and T/NK-LAHS for either of the two indicators (*p* > 0.05). Considering that macrophages/monocytes of sHLH produce a mixture of cytokines, particularly TNF and various interleukins (i.e., IL-6, IL-1β and IL-18), which trigger a series of inflammatory pathways and ultimately produce a cytokine storm (23), with elevated sCD25 as an early marker of T-cell activation in HLH and closely associated with IFN-γ (24). We believe that the normal range of sCD25 is <6400pg/ml, IL1RA is ≤206pg/ml, IFN-γ is ≤7pg/ml, and IL-18 is ≤50pg/ml. From **Table 2**, we can see that the above 4 indicators are obviously beyond the normal range. Therefore, as it was written in this essay, levels of sCD25, IL1RA, IFN-γ, and IL-18 were found to be significantly elevated in patients with NHL-LAHS, but no significant difference between B and T/NK (*p* > 0.05). It was hypothesized that all three cytokines were related to the inflammatory state of HLH itself and not much to the underlying disease (that is, lymphoma). A study also observed significant activation of the IL-18–IFN-γ axis in patients with sHLH/MAS, along with an increase in serum IL-1 receptor antagonist (IL-1Ra) (25), validating our speculation. Notably, this study found significantly higher IL-10 levels (median 55.35 pg/ml) in B-LAHS compared with other NHL-LAHS (*p* = 0.018). Ohno et al. studied 33 patients with LAHS and found significantly higher levels of IL-6, IL-10, and TNF-α in patients with B-LAHS than in patients with T/NK-LAHS, considering that these elevated cytokines were derived from the neoplastic B cells themselves (26). Previous studies found IP-10 elevating in HLH patients during the active phase and decreasing after chemotherapy and during recovery, and expression of IP-10 was enhanced in IFN-γ-activated macrophages in the liver, spleen, and bone marrow (27). It is suggested that IP-10 is associated with the pathophysiology of HLH. A retrospective study found higher levels of IP-10 in T/NK-LAHS patients than in B-LAHS patients (28), but in this study, IP-10 in NK/T-LAHS (median 275 pg/ml) was more significantly higher than other T-LAHS (median 95.15 pg/ml) (*p* = 0.008). Previously, IP-10 has been studied to play an important role in the pathogenesis of tissue necrosis and vascular injury associated with certain EBV-positive lymphoid tissue proliferation, such as EBV-positive lymphoma-like granuloma and ENKTL-NT (29). IP-10 was more significantly elevated in NK/T-LAHS than in other pathological types of NHL-LAHS in this study, considering that IP-10 is closely related to NK/T lymphoma.

Treatment of malignancy-triggered HLH needs to balance HLH-specific and tumor-specific treatment (30). In this study, the prognosis of LAHS patients who received HSCT was significantly better than that of patients who did not (*p* = 0.009). However, there was no significant difference in OS rate between patients receiving HSCT with LAHS and lymphoma (*p* = 0.200), suggesting that LAHS patients in HLH remission status can achieve the outcome of patients with lymphoma, so the treatment of LAHS patients should focus on HLH in the early stage and HLH remission lymphoma treatment afterward. The prognosis of NK-derived lymphoid proliferative diseases (LPD) with or without EBV differs significantly, the former exhibiting a more aggressive course (31). Since most quantitative polymerase chain reaction (PCR) assays target encapsulated viral particles and circulating EBV DNA, viremia may reflect only tumor-shed DNA and not viral replication (32). In this study, co-infection with EBV did not appear as an independent risk factor for poor prognosis in NHL-LAHS, which was considered to be influenced by EBV test and lymphoma subtype. Non-Hodgkin lymphoma can be divided into two groups, “indolent” and “aggressive,” based on the disease’s prognosis. The treatment of NHL varies greatly, depending on tumor stage, grade, and type of lymphoma, and various patient factors (e.g., symptoms, age, performance status) (33). For the former, high efficacy of first-line regimens in most patients with FL (CR rate is approximately 60%; range 40%–80%), and 10-year OS rate is up to 71% with many patients anticipating a normal life expectancy (34); for the latter, diffuse large B-cell lymphoma (DLBCL) is curable in about two-thirds of patients. Patients with germinal center B-cell-like (GCB) DLBCL have more favorable outcomes than those with activated B-cell-like (ABC) DLBCL when treated with standard immunochemotherapy, that is, R-CHOP (rituximab, cyclophosphamide, doxorubicin, vincristine, and prednisone) (35); a previous study confirmed that BL patients did not require stem cell transplantation and that disease-free survival (DFS) by R-HyperCVAD was comparable to the outcomes of other patients who received allo-HSCT (36). Aggressive PTCLs have a poor prognosis with most histologies having a 5-year survival of 30% with chemotherapy and are recommended consideration of ASCT for patients who achieve a CR or PR after high-dose chemotherapy (37). In this study, patients with NK/T-LAHS had a significantly worse prognosis than those with B-LAHS, and the pathological type of NK/T cell was found to be an independent risk factor for poor prognosis in patients with NHL-LAHS.

According to the epidemiological survey of HLH in China, the incidence of HLH in 2019 was 1.04/1,000,000, in which the incidence of LAHS was positively correlated with the age of onset (38). In this study, we mainly wanted to elucidate the clinical features of LAHS and its correlation with pathological types, and did not address the prevalence of LAHS; therefore, a large number of LAHS cases would be beneficial for our study. It is important to note that the data in this study came from a single-center, Capital Medical University affiliated Beijing Friendship Hospital, of which the hematology department is currently the largest HLH consultation and treatment center in China, with the highest annual intake of HLH patients in the country. This is why LAHS accounted for a large proportion of lymphoma patients in this study.

In the future, more cases and larger studies are needed to validate and revise the model, promoting the progress of LAHS research and updating the knowledge, assisting clinical decision-making, and improving patient prognosis.

## Data Availability Statement

The original contributions presented in the study are included in the article/supplementary material. Further inquiries can be directed to the corresponding authors.

## Ethics Statement

Written informed consent was obtained from the individual(s), and minor(s)’ legal guardian/next of kin, for the publication of any potentially identifiable images or data included in this article.

## Author Contributions

YW and ZW designed and performed research. YW, LH, RZ, ML, and ZH provided the materials and interpreted the data. SY and ZJ performed statistical analysis and wrote the manuscript. All authors contributed to the article and approved the submitted version.

## Funding

This work was supported by Beijing Municipal Administration of Hospital Clinical Medicine Development of Special Funding Support (XMLX201823) (collection of data and writing the manuscript); the National Natural Science Foundation of China (81871633) (analysis and interpretation of data); the Beijing Natural Science Foundation (7181003) (analysis and interpretation of data); and the Beijing Municipal Administration of Hospitals’ Ascent Plan (DFL20180101) (the design of the study).

## Conflict of Interest

The authors declare that the research was conducted in the absence of any commercial or financial relationships that could be construed as a potential conflict of interest.

## Publisher’s Note

All claims expressed in this article are solely those of the authors and do not necessarily represent those of their affiliated organizations, or those of the publisher, the editors and the reviewers. Any product that may be evaluated in this article, or claim that may be made by its manufacturer, is not guaranteed or endorsed by the publisher.
